# FODMAPs—Unknowns for Consumers: First Survey in Serbia

**DOI:** 10.3390/nu15214693

**Published:** 2023-11-05

**Authors:** Aleksandra Torbica, Vesna Vujasinović, Miloš Radosavljević, Goran Radivojević, Ilija Milovanović

**Affiliations:** 1 Institute of Food Technology, University of Novi Sad, Bulevar cara Lazara 1, 21102 Novi Sad, Serbia; 2 Faculty of Sciences, University of Novi Sad, Trg Dositeja Obradovića 3, 21000 Novi Sad, Serbia; vesna.vujasinovic@dgt.uns.ac.rs (V.V.); goran.radivojevic@dgt.uns.ac.rs (G.R.); 3 Faculty of Technology, University of Novi Sad, Bulevar cara Lazara 1, 21102 Novi Sad, Serbia; milosr@tf.uns.ac.rs; 4 Faculty of Philosophy, University of Novi Sad, Dr Zorana Đinđića 2, 21102 Novi Sad, Serbia; ilijamilovanovic@ff.uns.ac.rs

**Keywords:** wholegrain cereal products, gastrointestinal disorders, fructans, survey, consumer’s awareness

## Abstract

According to unofficial data, every fifth person in Serbia suffers from some form of irritable bowel syndrome (IBS). Compounds classified as FODMAPs (Fermentable Oligo-, Di-, and Monosaccharides and Polyols) are newly found potential triggers of IBS and a number of associated gastrointestinal disorders. Cereals, predominantly in their wholegrain form, represent the key contributors to the high contents of FODMAPs in wholegrain (high-fiber) bakery products. The current work was structured in a way to systematically evaluate the consumer’s knowledge and preferences toward wholegrain and low-FODMAP bakery products. The questionnaire was filled out by 725 respondents, aged from 18 to 86 years. They were informed about the aim of the research and management of anonymous data. The present study is the first detailed survey in this region of Europe, aiming to improve the familiarity with and attitude toward FODMAPs and a low-FODMAP diet by analyzing the different dietary habits regarding wholegrain-cereal-based products among consumers of various ages, genders, places of residence, and education. The results suggest that the respondents are, to some degree, aware of the health benefits of consuming foods with high fiber content while indicating a low level of knowledge about FODMAP compounds and connected topics. Education about contemporary scientific findings and the potentially harmful effects of consuming FODMAP compounds for a population with gastrointestinal disorders and diseases will be imperative in the future.

## 1. Introduction

Bakery products are an important part of the human diet and play a significant role in food consumption all over the world. Bread and pasta, followed by cookies, cakes, and breakfast cereals, represent the majority of industrially consumed cereal products [1]. However, bakery production is not stable. It varies and constantly adapts to consumer demand, which is not always comprehensible and, more importantly, is difficult to predict [2]. Of note, wholegrain products are a significant part of these cereal products, which have been promoted in the last decade due to their positive effects on the improvement of population health connected to their high dietary fiber (DF) content [3].

FODMAP compounds are a heterogeneous group that comprise galacto-oligosaccharides (GOSs), fructo-oligosaccharides (FOSs), and fructan polysaccharides (with the last two making up total fructans), lactose, polyols (sorbitol, mannitol, etc.), and fructose. However, fructose is considered a FODMAP only when it is in excess compared to glucose due to the saturation of its active transportation, indicating that it is hardly absorbed and thus potentially quickly fermented in the large intestine. Interest in the dietary control and intake of FODMAP-rich products lies in the fact that they worsen the symptoms of several gastrointestinal conditions and disorders (irritable bowel disease, celiac disease, and Crohn’s disease) [4,5,6,7].

Procedures for the modification of DF content and reduction in FODMAP compounds content in bakery products include several biotechnological tools. Namely, these tools include the addition of enzymes (such as endogenous and exogenous forms) and microbial fermentation (in the form of yeast and/or sourdough fermentation) as part of the standard or modified bread-making process. Exogenous enzymes represent commercially available complexes classified as production aids, while endogenous forms are obtained via the controlled germination and sprouting of specific cereals. During germination and sprouting, various biochemical pathways are activated, resulting, among others, in the synthesis of enzymes for the hydrolysis of stored substrates (energy reserves). Recent findings support the necessity for the modification of total DF composition, in addition to the conversion of insoluble DF (IDF) to soluble DF (SDF) in bakery products, particularly products from wholegrain cereals, to make them appropriate for consumers with gastrointestinal conditions and disorders [8].

Food choices refer to the consumer’s attitudes and beliefs. Attitudes represent the feelings or affections toward a product’s attributes. Beliefs represent the cognitive knowledge of consumers that connects benefits, attributes, and observed objects. For an evaluation of food products, consumers have numerous product criteria at their disposal, resulting in the final decision as to whether the product satisfies their expectations and requirements. Consumers develop and adopt a specific attitude toward the product on the grounds of a complex perception of product characteristics. Perception represents the filter between the consumer’s internal (subjective) and external (objective) impulses emerging during an evaluation of the product. The consumer’s preferences, choices, and attitudes are defined by human perception or subjectivity [9].

Taking into account the novelty of a low-FODMAP diet and the insufficient knowledge about the potential effects of FODMAPs and their presence in various products, the current work was structured in a way to systematically evaluate the consumer’s knowledge and preferences toward wholegrain and low-FODMAP bakery products. The present study is funded by the Science Fund of the Republic of Serbia to contribute to the first project that deals with FODMAPs. The aim of the study is to improve the familiarity with and attitude toward FODMAPs and a low-FODMAP diet by analyzing the different dietary habits regarding wholegrain bakery and cereal products among consumers of various ages, genders, places of residence, and education. The benefits of certain bakery products, their attributes, and people’s consumption habits were analyzed in detail. Also, consumer preferences and attitudes toward different methods for reducing FODMAPs were examined in order to investigate the general acceptance of innovation in the traditional production process. The overall goal was to gain a “wider” picture, to identify targeted products, and to determine what is acceptable for and applicable to the modification of current bakery products (toward low FODMAP content) and the formulation of new low-FODMAP bakery products.

## 2. Materials and Methods

### 2.1. Respondents

The study was conducted from June to December 2022 using an online survey in Google Forms (invitations were distributed via social media and through the university and institute database emails) and on site. After excluding non-qualifying and incomplete surveys (67), the remaining 725 surveys were used for the analysis (91.5%), as the sample was considered representative.

The age of respondents ranged from 18 to 86 years, and due to the need for further applied analysis in this work, respondents were divided into three subgroups: 18–25 years, 26–45 years, and over 46 years. All respondents were informed about the aim of the research and the management of anonymous data. After examination of the research study protocol for the project entitled “Biotechnological tools for optimization of short and medium chain carbohydrates content in cereal-based food to prevent gastrointestinal disorders–GutFriendlyCarbs” and relevant annexes, submitted by Dr. Vesna Vujasinović, the Ethical Review Committee of the Faculty of Science Novi Sad of the University of Novi Sad concluded that there were no ethical objections to the execution of the research project.

### 2.2. Materials and Procedure

The questionnaire used in this study (File S1) was composed of several groups of questions, whose aim was to determine consumers’ knowledge about FODMAP foods and their impact on health, attitudes toward healthy cereal products (wholegrain flour products and products with reduced FODMAPs content), and habits related to the consumption and purchase of cereal-based products (bread, pasta, cookies, and cakes).

Sociodemographic variables: Questions referring to sociodemographic characteristics were placed in the first part of the questionnaire. Examinees were asked to provide answers about their gender, age, education level, place of residence, occupation, and monthly income.

The health impact of FODMAPs: This section consisted of 9 questions related to respondents’ knowledge about FODMAP compounds, their presence in certain foods, and their association with certain gastrointestinal disorders and diseases. The questions were formed to suit the needs of our research based on a literature review [5,7,10,11,12]. For the question “*Food that contains significant amounts of FODMAPs: (different foods are listed)*”, respondents were asked to evaluate the level of their agreement with the provided statements on a 5-point Likert scale (1—completely disagree; 5—completely agree).

Attitudes regarding cereal-based products: This part of the questionnaire was based on a survey conducted by Dean et al. [13], which aimed to explore people’s perceptions of healthy cereal products (cholesterol-lowering and high-fiber products) and production methods. Some items were adapted in order to enable research on consumers’ perceptions of products with reduced FODMAPs content, which have yet to be introduced to the market, and wholegrain flour products, which are already present in the Serbian market. All items were back-translated into Serbian by the authors, and the accuracy of the translation was verified. To assess the level of agreement, respondents marked answers from 1 (strongly disagree) to 7 (strongly agree) (7-point Likert scale). Perceptions of benefits and willingness to use were measured with the questions “Eating cereal products with reduced FODMAPs content/wholegrain flour would be beneficial to me” and “Would you be willing to use cereal products with reduced FODMAPs content/wholegrain flour?”, respectively, for all four types of cereal products: bread, pasta, cookies, and cake.

Attitudes toward five different processing methods were measured on a seven-point scale, ranging from negative (disagreement) to positive (agreement with a statement). The methods offered for FODMAPs reduction were the use of commercial purified enzymes from microorganisms (1), the use of various plant extracts (2), the use of germinated grains (3), the application of a modified standard production process with yeast (4), and the application of a modified traditional process of sourdough production with lactic acid bacteria (LAB) (5). These methods represent biotechnological processes that use GRAS-approved additives and microorganisms, and many of them are already part of the standard bread-making process. Commercial enzymes or endogenous enzymes (added as part of germinated grains) are excellent substitutes for chemical additives, hydrocolloids, and emulsifiers. Enzyme addition is advantageous because it can target specific FODMAP representatives in staple bakery products, and the enzyme has no activity after baking, thus helping in the development of clean-label products. Microbial fermentation, a traditional process using yeast and/or LAB, besides representing a well-known contribution to the formation of bakery products, also reduces the content of FODMAP compounds (FOS and GOS) during bread making [14,15]. Even before the development of enzymology, some plant parts and their extracts were used in a number of traditional processes, like meat tenderization using papaya leaves, curd and cheese making, soy sauce preparation, baking, and brewing processes. Plant extracts (obtained using “green” techniques) containing FODMAP-degrading enzymes can be obtained by using specific plants that are rich in FODMAP precursors and/or compounds because they have enzymes responsible for their formation and degradation during plant growth. [16].

Participants’ attention to dietary health concerns was assessed by their responses to four questions that allowed them to indicate on a 7-point scale (from strongly disagree to strongly agree) how much attention they felt they needed to pay to specific health issues. Questions on health issues included attitudes toward overall dietary health, fiber intake, gut health, and digestive problems.

Participants’ self-perception of health was measured with one item, “I consider myself as a health-conscious person”, on a 7-point scale.

The importance of certain characteristics (the price, taste, health impact, appearance/texture, being new, being traditional) when choosing/purchasing bread, pasta, cookies, and cakes was measured on a 6-point scale (1—very unimportant; 6—very important) adopted from Bruschi et al. [17]

Habits regarding the purchase of cereal-based products: The third part of the questionnaire included a series of multiple-choice questions about the amount and frequency of consumption of cereal products, the type of flour and processing method used for cereal product preparation, and the place of purchase. The questions were formed for the needs of our research based on a literature review [18,19] considering the gastronomic culture and tradition.

Respondents were given the opportunity to comment, especially on the group of questions about the health impact and habits related to the consumption and purchase of cereal products.

### 2.3. Data Analysis

Statistical analysis of the collected data was carried out using Statistica R 4.2.1. Descriptive statistics are used to present sociodemographic characteristics, the knowledge of FODMAPs, and habits related to the consumption and purchase of cereal products, and data are presented in frequency tables.

Correlation analysis was performed to examine the relationship between the items of attention to dietary health concerns (Pearson’s coefficients at *p* < 0.05 and *p* < 0.01 were examined). *t*-tests were performed to examine the differences between genders and a low/high need to pay attention to health in terms of perceived benefits. ANOVAs were conducted to examine differences between age and methods used for FODMAPs content reduction in cereal products. A criterion of *p* < 0.01 or *p* < 0.05 was chosen.

## 3. Results

### 3.1. Sociodemographic Characteristics

The sample comprises a wide range of consumers regarding sociodemographic characteristics (Table 1). Noticeably, there is a slightly higher proportion of female respondents in the survey, which is consistent with the differences in the proportion of genders in the population of R. Serbia, according to the 2022 census [20], with 51.3% women and 48.7% men. It also indicates that women were more willing to participate in the survey.

According to the 2022 census [20], the average age of the population of the Republic of Serbia is 43.8 years. The mean of 37.3 years (S.D. = 15.3) of the sample indicates a slight undersampling of the elderly compared to the average age of the population.

Data of interest for further analysis of the results obtained in this research are that 75.3% of respondents stated they had never heard of FODMAPs, and 13.4% said that they knew something about them but were unaware of their health effects.

### 3.2. Frequency of the Consumption of Certain Cereal Products

The Republic of Serbia is a Balkan country with a long tradition of agricultural production and a high contribution of cereal-based products and foods (such as wheat flour and bread, pasta, and bakery products) to nutrition [18,21,22], with an average daily consumption of 166 g of bread, 27 g of pasta, and 19 g of cookies per capita [19].

The analysis of the data collected in the survey showed that cereal-based products make up a significant portion of the nutrition of the population of Serbia, as 76.1% of respondents stated that they consume bread, 58.5% consume pasta, and 37.9% consume breakfast cereals. A total of 39.3% of respondents buy bread on a daily basis, with a dominant share of 75% being wheat bread. The frequency of consumption of rye bread is 33.9%, while corn and buckwheat bread are consumed at 22.5% and 17.8%, respectively. White wheat bread is reported to be the most consumed type of bread (60%), followed by wholegrain bread (28.3%). Most respondents consume bread made by a standard production process with yeast (73.5%), followed by plain bread (without yeast) (17.8%); soda bread (6.2%), bread with germinated grains (5.1%), and sourdough bread (4.3%) are also considered important to mention.

The majority of respondents (52.3%) consume pasta on a monthly basis and, similar to bread, 71.3% of consumers prefer pasta made from wheat flour, while pasta made from buckwheat flour and spelt flour are less prevalent at 10.2% and 6.1%, respectively. As with bread, pasta made from white flour is the most commonly consumed type (77.4%), while the consumption of wholegrain pasta is slightly lower compared to wholegrain bread consumption (24%).

Regarding cookies, a similar percentage of respondents, 34.8% and 33.7%, consume cookies on a weekly and monthly basis, respectively. The most consumed cookies are made of white wheat flour (68.5%), while the consumption of wholegrain cookies is as high as that of bread, 28%. The majority of respondents consume cookies made from wheat flour (71.8%), and in contrast to other cereal products, the preference for oat flour is highest (16%).

As far as confectionery (cakes) is concerned, the majority of respondents consume them on a monthly basis (45.2%), while 23.9% and 16.1% consume them on a weekly and annual basis, respectively. Unlike other cereal products, one-third of respondents report making cakes themselves rather than buying them.

Cakes made from white wheat flour are the most consumed (77.8%), while wholegrain cakes are the least consumed compared to other cereal products (13.1%).

### 3.3. Assessment of Health Impact and Knowledge about FODMAPs

The increasingly frequent occurrence of food allergies worldwide [23,24], in association with poor digestion/nutritional intake and intestinal health disorders [25,26,27], made it necessary to investigate the health status of the respondents. According to the results of the conducted research, 43% of respondents reported having health problems associated with eating disorders, of which only 4.3% have a medically confirmed diagnosis, while 7.4% of the respondents are still in the phase of medical examination.

**Figure 1:**
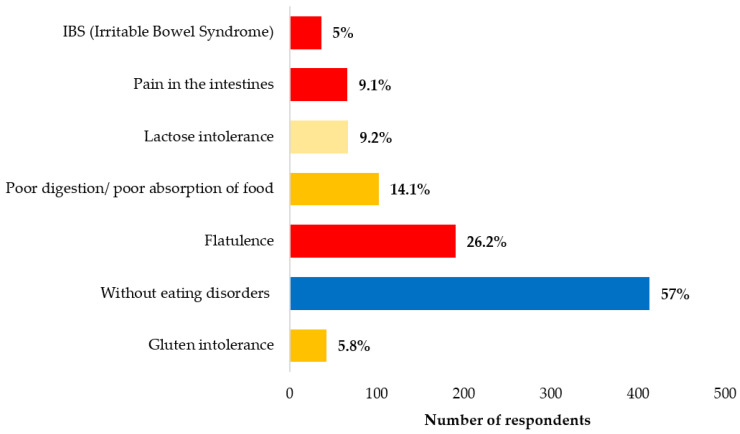
Share of health problem occurrences among respondents (*N* = 725).

The most frequently mentioned health disorders that may be associated with the presence of FODMAPs in foods include flatulence, suffered by 26.2% of respondents; intestinal pain, experienced by 9.1% of respondents; irritable bowel syndrome (IBS) (5%); Crohn’s disease (0.6%); and celiac disease (0.4%) (Figure 1) [26,28,29,30].

Therefore, the next question of interest was to determine the participants’ knowledge of FODMAPs and their contents in particular foods (Table 2).

It can be seen that the majority of respondents do not have a clear idea of the possible sources of FODMAPs in the diet, as the mean values for FODMAPs content perception for most foods are around 3. This means that the respondents neither agree nor disagree with the claim that the mentioned foods contain significant amounts of FODMAP compounds. According to the results, food products considered to have high FODMAPs content are mainly candies, chocolate, cookies, and cakes. These are food products that are commonly considered to be rich in carbohydrates. Other foods with high FODMAPs content, according to the respondents, are cereals and bakery products and alcoholic beverages. On the other hand, the foods that respondents believe contain fewer of these compounds are fish, seafood, meat, and meat products. However, it must be noted that respondents did not recognize legumes as foods with high FODMAPs content (predominantly GOS) and their connection to intestinal health disorders. The targeted high-FODMAP-content foods are wholegrain products (0.6–6.6%), legumes (0.6–7.6%), and fruits and vegetables (0.6–2.7%) [31,32,33].

### 3.4. Healthy Cereal Product Preferences

In recent decades, based on epidemiological studies, there has been some evidence that the regular consumption of wholegrain products leads to a reduction in the incidence of certain diseases, such as type 2 diabetes [34], coronary heart disease [35], and certain cancers [36]. Wholegrain products, in the form of both staple foods such as bread and pasta and hedonistic foods such as cookies, have a positive effect on human health and are widely available in the domestic market of the Republic of Serbia. Also, it can be observed that 28.3% of respondents consume bread made from whole grains, while 24% consume wholegrain pasta, providing evidence that at least 25% of respondents have developed an awareness of the health benefits of wholegrain products.

Considering the consequences and health problems that FODMAPs can cause in the body [10,37,38], the introduction of cereal products with reduced FODMAPs content to the domestic market can be beneficial. This is especially significant because 75% and 33.9% of respondents consumed bakery products made from wheat and rye, respectively. For this reason, in the continuation of the research, the perceptions and preferences for these two types of products were compared.

Analyzing the responses to the questions on attitudes regarding attention to dietary health concerns, a strong correlation was found between these four items. A composite score using four attention-to-dietary-health-concerns measures was calculated and labeled “health awareness” (Cronbach’s α = 0.876). After applying a correlation analysis, the results showed that health awareness was significantly positively correlated with participants’ self-perception as a health-conscious person (r = 0.48; *p* < 0.05; n = 724), which can be interpreted as individuals who perceive themselves as health-conscious also having a high need to take care of their health and vice versa. To determine the difference in the perceptions of benefits, *t*-tests were carried out.

According to the obtained results (Table 3), respondents considered staple wholegrain products (bread and pasta) to be significantly more beneficial than hedonistic wholegrain foods (cookies and cakes), which is consistent with the findings of Shepherd et al. [39]. There are no statistically significant differences in cereal products with reduced FODMAPs content. The respondents consider wholegrain bread and wholegrain pasta significantly more beneficial than the same products with reduced FODMAPs content (Table 3).

It can be concluded that consumers have certain information and are aware of the health benefits of consuming foods with high fiber content, while results indicate a low level of knowledge about FODMAP compounds. For this reason, it is necessary to educate them about contemporary scientific findings and the potentially harmful effects of consuming FODMAP compounds in order to change their attitudes toward and perceptions of the health benefits of low-content FODMAP foods. This is especially important since gastrointestinal conditions and disorders affect 6.5 to 34.2% of the global population, with an increased prevalence in women [11,40,41].

### 3.5. Differences between Genders, Health Attention Needs, and Ages in the Perception of the Benefits of Certain Cereal Products

In order to perform the next analysis, respondents were divided into two groups based on the median need to pay attention to dietary health concerns (median = 5)—those with a low need (n = 424, 58.5%) and those with a high need to attend to their health (n = 301, 41.5%).

A *t*-test was conducted to examine the influence of gender and a low/high need to pay attention to health on the perceived benefits of healthy cereal products.

The results presented in Table 4 show that women perceived more benefits from all types of wholegrain cereal products, as well as cookies and cakes, with reduced FODMAPs content compared to men. In addition, respondents with a high need to pay attention to their health saw significantly more benefits in all types of healthy cereal products, except for wholegrain cakes. The obtained results presented in Table 4 indicate that respondents aged 46+ perceive more benefits from all wholegrain products, except for bread.

To further examine and confirm the above assumptions, the answers to health attention questions (overall dietary health, fiber intake, gut health, and digestive problems) were correlated with age, residence, and education. The correlation results suggest that with an increase in age, people pay more attention to digestive problems (positive correlation, *p* < 0.05). Residence had a strong correlation (positive *p* < 0.01) with overall dietary health and fiber intake, meaning that people from more inhabited areas pay more attention to novel dietary practices and trends. The level of education had a strong positive correlation (*p* < 0.01) with answers to all health attention questions, providing evidence that the higher the education, the more people aspire to a healthier lifestyle.

### 3.6. Willingness to Buy Specific Healthy Grain Products

An examination of the relationship between respondents’ perceived benefits of specific healthy cereal products and their willingness to purchase them revealed that for all products (bread, pasta, cookies, and cakes), there was a moderate positive correlation for both wholegrain products and products with a reduced FODMAPs content, with correlation coefficients ranging from 0.44 to 0.59, except for wholegrain bread, where the correlation coefficient was strong, r = 0.6. This implies that those who perceived greater benefits were moderately willing to purchase these products since the price of the product also affects the willingness to acquire it. The price of common white wheat bread is 2 and 2.5 times lower than the price of wholegrain bread and special kinds of bread, respectively [42].

### 3.7. Preferences toward Different Product Attributes and Consumption Habits

In order to determine the importance of various product attributes (price, taste, health effects, appearance/texture, novelty, and being traditional) when purchasing certain cereal products (bread, pasta, cookies, and cakes), repeated-measures ANOVA and the Bonferroni test (correction) were carried out (see Table 5).

The results show that there are statistically significant differences between attributes for specific products, with taste standing out as the most preferable one for all groups of products. The positive impact of cereal products on health, even hedonistic products like cookies and cakes, is considered a very important attribute, which is consistent with the findings of the work mentioned above that consumers are “health conscious”, and it represents a more important attribute than the price [17]. The attribute that, according to the respondents, is least important when purchasing cereal products is novelty (being a new product) in the market, especially for staple products such as bread and pasta.

Women considered the health effects of all cereal products to be significantly more important than men did, as well as the appearance/texture of the product (Table 6).

Women also considered the taste of cookies and cakes to be more important than men did when purchasing certain cereal products. To further analyze the preferences toward different product attributes, they were correlated with age, residence, and education. A strong positive correlation (*p* < 0.01) regarding age was observed only with the price aspect of the bread, while a negative correlation was observed for the appearance/texture of the bread (*p* < 0.05). This means that the older generation sees the price as the limiting factor for the acquisition of bread, while the younger generation uses appearance/texture as a decisive factor when purchasing bread. Residence had a strong correlation (positive *p* < 0.01) only with the novelty aspect of cookies, meaning that people from more inhabited areas have a higher regard for novelty when purchasing cookies. The level of education had a strong positive correlation (*p* < 0.01) with price, providing evidence that the higher the education, the more attention paid to the prices of bread and cookies.

### 3.8. Attitudes toward Methods Used for FODMAPs Reduction

Five different methods for FODMAPs content reduction in cereal products were proposed in the questionnaire: reduction with the use of purified commercial enzymes from microorganisms, the use of various plant extracts, the use of germinated grains, the application of a modified standard production process with yeast, and the application of a modified traditional process of sourdough production with lactic acid bacteria.

**Figure 2:**
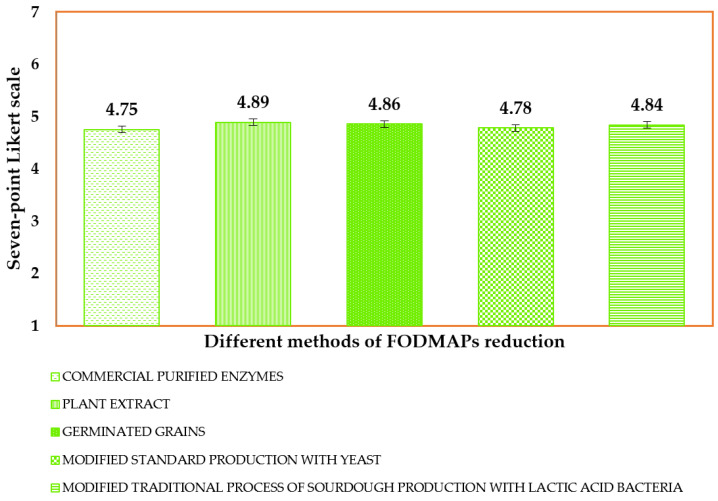
Preferences regarding different methods of FODMAPs reduction (means and standard errors).

Comparing the obtained mean values using ANOVA (F (4) = 0.95, *p* = 0.44) (Figure 2), it can be seen that there is no statistically significant difference regarding the preferences for different FODMAPs reduction methods.

For further analysis, differences in preferences depending on gender and age were observed. According to the results (Table 7), women were significantly more positive than men regarding all proposed methods for FODMAPs content reduction, except for the modified standard production process with yeast. This is expected since the production process with yeast is a well-known procedure. The female respondents had more pronounced attitudes toward all proposed methods for FODMAPs content reduction.

ANOVA was conducted to compare differences related to age (Table 8). The only statistically significant differences with respect to age were found when different plant extracts were proposed for FODMAPs content reduction, in the sense that respondents aged 26–45 years were more positive about this method of reduction than respondents aged over 46 years. The results suggest that the population aged 18–25 is indifferent to novelty in the production process, while the population aged 46+ prefers traditional methods. The population aged 26–45 prefers novelty and is most susceptible to potential changes in the diet. Thus, the efforts toward education must be directed to age groups below 26 and above 45.

## 4. Discussion

Today, insight into the preference for and consumption of specific staple foods is almost mandatory in formulating particular food products. Regardless of the fact that the low-FODMAP diet was first described two decades ago [12], this study showed that the majority of respondents in Serbia did not know about FODMAPs or about their presence in foods with this defined terminology. Even though the FODMAPs terminology was explained in the introduction of the survey, the majority of respondents did not connect their presence to specific foods, meaning that the potential (average) consumer does not have a clear picture of what high-FODMAP-containing foods are nor recognize legumes as such foods in addition to wholegrain products, fruits, and vegetables [31]. Based on the responses and a self-assessment, a significant number of respondents have some form of gastrointestinal condition or disorder that may be associated with the presence of FODMAPs in food. Not having a clear picture of the presence of FODMAPs in certain foods and their potentially negative health impacts, respondents were not able to clearly distinguish between the health benefits of wholegrain products and those of products with reduced FODMAPs content, considering the wholegrain products to be the food providing more health benefits. This could be because the public is more familiar with fiber-added grain products being recognized as functional food [43] than low-FODMAP products, which have yet to be introduced to the market of the Republic of Serbia.

Further results showed that consumers considered staple wholegrain products (bread and pasta) to be significantly more beneficial than wholegrain hedonistic ones, which is in line with findings from the previous study by Shepherd et al. [39] and the fact that people have been found to ignore nutritional information on hedonistic foods [44]. The low level of knowledge about FODMAP compounds and their potentially harmful effects indicates the necessity for educating the public about contemporary scientific findings, especially since gastrointestinal conditions and disorders affect one-third of the global population.

As expected, those who said that they need to pay attention to their health perceived more benefits from all types of healthy cereal products, except for wholegrain cakes. According to the results, respondents aged 46+ perceive more benefits from all wholegrain products, except for bread, which is consistent with the fact that with an increase in age, people pay more attention to digestive problems.

The analysis of preferences for different product attributes and consumption habits for bread and cookies (as the most frequently purchased cereal-based products) showed that older generations and those with a higher level of education see the price of the product as a limiting factor, while the younger generation favors appearance/texture during the acquisition of bread. Taste is the most preferable attribute for all groups of products, with women considering it to be more important than men do. The positive impact of cereal products on health, even hedonistic products like cookies and cakes, is considered a very important attribute and consistent with the findings of the work showing that consumers are considered “health conscious”. In accordance with the findings of Bruschi et al. [17], taste represents a more important attribute than price; thus, it must be taken into account when introducing a new product to the market. People from more inhabited areas have a higher regard for novelty when purchasing cookies.

Answers addressing attitudes toward specific methods used for FODMAPs reduction, namely, reduction with the use of purified commercial enzymes from microorganisms, the use of various plant extracts, the use of germinated grains, the application of a modified standard production process with yeast, and the application of a modified traditional process of sourdough production with lactic acid bacteria, as is currently applied on the global market [12], showed no statistically significant differences regarding Serbian consumers’ preferences for different FODMAPs reduction methods. This leaves at our disposal the possibility for the optimization and application of all mentioned methods for FODMAPs reduction in order to achieve the most favorable sensorial properties. Further analysis of the differences in preferences depending on age suggests that the population aged 18–25 is indifferent to novelty in the production process, and the population aged 46+ prefers traditional methods, while the population aged 26–45 prefers novelty and is most susceptible to potential changes in the diet.

As the bottom line, the respondents have certain information and are aware of the health benefits of consuming foods with high fiber content, while the results indicate a low level of knowledge about FODMAP compounds and connected topics. For this reason, it is necessary to educate consumers about contemporary scientific findings and the potentially harmful effects of consuming FODMAP compounds in order to change their attitudes toward and perceptions of the health benefits of low-content FODMAP foods. It is suggested that targeted and specific FODMAP topics be explained both online and in peer groups in order to improve the knowledge and attitudes toward specific FODMAP foods and diets to positively influence their food practices. The results of this study will greatly aid the authors in defining the procedures and formulation of low-FODMAP bakery products, which have yet to be presented to the Serbian market. Moreover, as the results suggest, these low-FODMAP wholegrain products must be acceptable in both taste and price aspects, which is quite challenging from technological and production points of view.

## 5. Conclusions

This is the first study in Serbia regarding the FODMAP-related topic. The main result of the survey is the almost complete ignorance of FODMAP compounds and the lack of information on the types of foods in which they are found in large quantities. This imposes the need to declare such foods so that the population with health problems worsened by the consumption of FODMAP compounds can avoid such foods when buying. Before that, it is necessary that decision-makers make the determination of total FODMAP compounds in products based on whole grains and legumes mandatory, as well as define the cutoff values for specific FODMAP compounds and total FODMAP content. Furthermore, the above-mentioned activities must be accompanied by the education of the general population about FODMAPs and the foods that contain them, while nutritionists should offer consumers with gastrointestinal disorders examples of balanced diets for the highest possible quality of life. Researchers are tasked with finding as many ways as possible to reduce FODMAPs so that consumers have a choice of low-FODMAP products and the ways in which they are reduced according to their own preferences. The consumption of low-FODMAP products is a unique solution for simultaneous high dietary fiber intake and the replacement of nutritionally imbalanced low-FODMAP diets.

## Figures and Tables

**Table 1:** Sociodemographic characteristics of the sample (n and % of respondents, *N* = 725).

		n	%
Gender	Male	284	39.2
	Female	432	59.6
	Other	9	1.2
Age	Mean (S.D.)	37.3 (15.3)	
	18–25 years	233	31.6
	26–45 years	276	37.4
	≥46 years	216	29.3
**RESIDENCE**	Larger urban area	409	56.4
	Smaller urban area (<100,000 inhabitants)	185	25.5
	Village	131	18.1
Education	Elementary school	12	1.7
	High school	342	47.2
	Faculty	225	31
	MSc/PhD	146	20.1

**Table 2:** Differences in perceptions of FODMAPs content in certain foods (*N* = 725).

	Negative ^a^ (%)	Neutral ^b^ (%)	Positive ^c^ (%)	Mean	SE
Milk and dairy products	20	43.3	36.7	**3.15**	0.034
Meat and meat products	39.2	39	21.8	**2.73**	0.037
Fruits	26.4	33.4	40.2	**3.14**	0.042
Nuts	34.1	39.3	26.6	**2.87**	0.038
Vegetables	35.4	39.9	24.7	**2.83**	0.039
Legumes	28.4	40.4	31.2	**2.97**	0.039
Fish and seafood	46.3	36	17.7	**2.58**	0.038
Cereals and bakery products	12.3	30.9	56.8	**3.61**	0.038
Spices	29	41.5	29.5	**2.97**	0.039
Alcoholic beverages	19.8	29.4	50.8	**3.43**	0.044
Chocolate	13.9	26.2	59.9	**3.67**	0.042
Candies	12.8	26.9	60.3	**3.7**	0.043
Cookies	12.4	25.4	62.2	**3.75**	0.042
Cakes	11.8	23.4	64.8	**3.79**	0.043

SE—standard error. ^a^ Category “negative” includes “strongly disagree” and “disagree” from the 5-point Likert scale, which have been merged for clarity of presentation. ^b^ Category “neutral” is “neither agree nor disagree” from the 5-point Likert scale. ^c^ Category “positive” includes “agree” and “strongly agree” from the 5-point scale, which have been merged for clarity of presentation.

**Table 3:** Differences in perceived benefits of wholegrain products and products with a reduced FODMAPs content (means and standard errors) *.

Benefit Perception	Bread	Pasta	Cookies	Cakes	*p*
**Wholegrain products**	5.39 ^bB^ (0.059)	5.32 ^bB^ (0.059)	5.14 ^aA^ (0.062)	5.11 ^aA^ (0.064)	Bread, pasta > cookies, cakes **0.04**
**Low-FODMAP-content products**	5.08 ^aA^ (0.059)	5.10 ^aA^ (0.060)	5.10 ^aA^ (0.060)	5.14 ^aA^ (0.060)	No difference 0.68
***p***	<0.001	<0.001	0.30	0.40	

* All variables were compared by employing ANOVA for dependent measures. Means sharing a superscript small letter within a row are not significantly different (*p* > 0.05); Means sharing a superscript capital letter within a column are not significantly different (*p* > 0.05).

**Table 4:** Differences between genders, ages, and those with low/high need to attend to health regarding benefit perceptions relating to certain cereal products (means and standard errors).

Bread—WHOLEGRAIN (F = 0.81)				
Gender	Mean	Gender	*p*	Standard Error
Males	5.12	Female	**<0.001**	0.099
Females	5.56	Female	-	0.073
**Age**	**Mean**	**Age**	***p***	**Standard Error**
18–25	5.49	26–45	1.00	0.141
		46+	0.627	0.150
26–45	5.37	46+	1.00	0.144
46+	5.30	46+	-	-
**Attention to health**	**Mean**	**Attention to health**	***p***	**Standard Error**
Low need to pay attention to health	5.19	High need to pay attention to health	**<0.001**	0.078
High need to pay attention to health	5.67	High need to pay attention to health	-	0.087
**Bread—Low-FODMAP (F = 2.08)**				
**Gender**	**Mean**	**Gender**	***p***	**Standard Error**
Males	4.93	Female	0.04	0.093
Females	5.17	Female	-	0.076
**Age**	**Mean**	**Age**	***p***	**Standard Error**
18–25	4.95	26–45	0.153	0.141
		46+	1.000	0.150
26–45	5.23	46+	0.490	0.144
46+	5.03	46+	-	-
**Attention to health**	**Mean**	**Attention to health**	***p***	**Standard Error**
Low need to pay attention to health	4.83	High need to pay attention to health	**<0.001**	0.077
High need to pay attention to health	5.43	High need to pay attention to health	-	0.088
**Pasta—WHOLEGRAIN (F = 0.14)**				
**Gender**	**Mean**	**Gender**	***p***	**Standard Error**
Males	4.93	Female	**0.004**	0.099
Females	5.30	Female	-	0.080
**Age**	**Mean**	**Age**	***p***	**Standard Error**
18–25	5.30	26–45	1.00	0.142
		46+	1.00	0.150
26–45	5.29	46+	1.00	0.145
46+	5.37	46+	-	-
**Attention to health**	**Mean**	**Attention to health**	***p***	**Standard Error**
Low need to pay attention to health	4.96	High need to pay attention to health	<0.001	0.080
High need to pay attention to health	5.41	High need to pay attention to health	-	0.096
**Pasta—Low-FODMAP (F = 1.20)**				
**Gender**	**Mean**	**Gender**	***p***	**Standard Error**
Males	5.00	Female	0.203	0.096
Females	5.16	Female	-	0.078
**Age**	**Mean**	**Age**	***p***	**Standard Error**
18–25	5.01	26–45	0.461	0.143
		46+	1.000	0.152
26–45	5.22	46+	0.727	0.146
46+	5.05	46+	-	-
**Attention to health**	**Mean**	**Attention to health**	***p***	**Standard Error**
Low need to pay attention to health	4.90	High need to pay attention to health	**<0.001**	0.077
High need to pay attention to health	5.39	High need to pay attention to health	-	0.093
**Cookies—WHOLEGRAIN (F = 0.78)**				
**Gender**	**Mean**	**Gender**	***p***	**Standard Error**
Males	4.90	Female	**0.008**	0.096
Females	5.22	Female	-	0.077
**Age**	**Mean**	**Age**	***p***	**Standard Error**
18–25	5.16	26–45	1.000	0.148
		46+	1.000	0.158
26–45	5.05	46+	0.657	0.152
46+	5.24	46+	-	-
**Attention to health**	**Mean**	**Attention to health**	***p***	**Standard Error**
Low need to pay attention to health	4.78	High need to pay attention to health	**<0.001**	0.078
High need to pay attention to health	5.55	High need to pay attention to health	-	0.086
**Cookies—Low-FODMAP (F = 0.01)**				
**Gender**	**Mean**	**Gender**	***p***	**Standard Error**
Males	4.90	Female	**0.008**	0.096
Females	5.22	Female	-	0.077
**Age**	**Mean**	**Age**	***p***	**Standard Error**
18–25	5.11	26–45	1.00	0.144
		46+	1.00	0.153
26–45	5.10	46+	1.00	0.147
46+	5.09	46+	-	-
**Attention to health**	**Mean**	**Attention to health**	***p***	**Standard Error**
Low need to pay attention to health	4.78	High need to pay attention to health	**<0.001**	0.078
High need to pay attention to health	5.55	High need to pay attention to health	-	0.086
**Cakes—WHOLEGRAIN (F = 1.32)**				
**Gender**	**Mean**	**Gender**	***p***	**Standard Error**
Males	4.87	Female	**0.002**	0.103
Females	5.28	Female	-	0.083
**Age**	**Mean**	**Age**	***p***	**Standard Error**
18–25	5.06	26–45	1.000	0.154
		46+	0.399	0.144
26–45	5.04	46+	0.544	0.157
46+	5.27	46+	-	-
**Attention to health**	**Mean**	**Attention to health**	***p***	**Standard Error**
Low need to pay attention to health	4.98	High need to pay attention to health	0.12	0.083
High need to pay attention to health	5.31	High need to pay attention to health	-	0.101
**Cakes—Low-FODMAP (F = 0.23)**				
**Gender**	**Mean**	**Gender**	***p***	**Standard Error**
Males	4.94	Female	**0.009**	0.095
Females	5.26	Female	-	0.079
**Age**		**Age**	***p***	**Standard Error**
18–25	5.19	26–45	1.00	0.144
		46+	1.00	0.153
26–45	5.10	46+	1.00	0.147
46+	5.12	46+	-	-
**Attention to health**	**Mean**	**Attention to health**	***p***	**Standard Error**
Low need to pay attention to health	4.91	High need to pay attention to health	**<0.001**	0.077
High need to pay attention to health	5.45	High need to pay attention to health	-	0.094

*t*-test for independent samples was used to test the gender differences and differences between low and high need to pay attention to health. Age differences were compared by employing ANOVA.

**Table 5:** Importance of different product attributes when purchasing (means and std. error, *N* = 725) *.

	Bread	Pasta	Cookies	Cakes
**Price**	3.62 ^bc^ (0.060)	3.73 ^c^ (0.059)	3.65 ^c^ (0.060)	3.68 ^c^ (0.062)
**Taste**	4.43 ^e^ (0.062)	4.43 ^e^ (0.059)	4.45 ^e^ (0.065)	4.58 ^e^ (0.065)
**Health effect**	4.14 ^d^ (0.058)	4.06 ^d^ (0.056)	3.99 ^d^ (0.058)	3.95 ^d^ (0.059)
**Appearance/texture**	3.84 ^c^ (0.058)	3.81 ^c^ (0.057)	3.83 ^cd^ (0.060)	4.01 ^d^ (0.062)
**Novelty**	2.99 ^a^ (0.058)	3.01 ^a^ (0.058)	3.12 ^a^ (0.059)	3.19 ^a^ (0.061)
**Traditional product**	3.46 ^b^ (0.059)	3.33 ^a^ (0.058)	3.32 ^b^ (0.059)	3.39 ^b^ (0.062)
**F**	58.05	61.05	55.65	61.10

All variables were compared by employing ANOVA. * Means sharing the same small letter in superscripts within a column are not significantly different (*p* > 0.01).

**Table 6:** Gender differences in preferences toward different product attributes (means, *N* = 725) *.

	Price	Taste	Health Effect	Appearance/Texture	Novelty	Traditional Product
**Bread**						
**Males**	3.64 (0.098)	4.30 (0.102)	3.93 * (0.094)	3.62 * (0.098)	2.91 (0.094)	3.39 (0.099)
**Females**	3.61 (0.078)	4.51 (0.079)	4.32 * (0.074)	3.97 * (0.072)	3.05 (0.075)	3.53 (0.075)
***p***	0.83	0.14	**0.01**	**0.01**	0.32	0.32
**Pasta**						
**Males**	3.67 (0.093)	4.32 (0.095)	3.89 * (0.091)	3.64 * (0.092)	2.95 (0.091)	3.22 (0.095)
**Females**	3.78 (0.077)	4.50 (0.076)	4.19 * (0.071)	3.91 * (0.072)	3.04 (0.075)	3.44 (0.075)
***p***	0.44	0.21	**0.04**	**0.05**	0.53	0.12
**Cookies**						
**Males**	3.56 (0.095)	4.28 * (0.105)	3.75 * (0.094)	3.58 * (0.094)	3.05 (0.093)	3.23 (0.095)
**Females**	3.71 (0.078)	4.58 * (0.082)	4.16 * (0.073)	4.05 * (0.077)	3.17 (0.077)	3.40 (0.075)
***p***	0.29	**0.04**	**0.01**	**0.01**	0.40	0.23
**Cakes**						
**Males**	3.55 (0.099)	4.36 * (0.109)	3.74 * (0.096)	3.73 * (0.099)	3.21 (0.096)	3.27 (0.101)
**Females**	3.77 (0.081)	4.74 * (0.080)	4.11 * (0.075)	4.21 * (0.079)	3.20 (0.080)	3.48 (0.079)
***p***	0.12	**0.01**	**0.01**	**0.01**	0.98	0.14

*t*-test for independent samples was used to test the gender differences. * Means marked with asterisk within a column are significantly different (*p* < 0.05).

**Table 7:** Gender differences in preference relating to different methods used for FODMAPs content reduction (means and standard errors) *.

	Commercial Purified Enzymes from Microorganisms	Different Plant Extracts	Germinated Grains	Modified Standard Production Process with Yeast	Modified Traditional Process of Sourdough Production with Lactic Acid Bacteria
**Males**	4.52 (0.099)	4.67 (0.096)	4.64 (0.099)	4.62 (0.103)	4.68 (0.103)
**Females**	4.90 (0.073)	5.04 (0.076)	5.01 (0.080)	4.89 (0.083)	4.96 (0.083)
***p***	**0.01**	**0.01**	**0.01**	0.06	**0.05**

*t*-test for independent samples was used to test the gender differences. * Means sharing a superscript within a column are not significantly different (*p* > 0.05).

**Table 8:** Age differences in preference relating to different methods used for FODMAPs content reduction *.

	Age				
	18–25	26–45	≥46	F	*p*
**Commercial purified enzymes from microorganisms**	4.77	4.89	4.56	2.29	0.19
**Different plant extracts**	4.90 ^ab^	5.06 ^a^	4.68 ^b^	3.58	**0.04**
**Germinated grains**	4.76	5.01	4.78	1.61	0.31
**Modified standard production process with yeast**	4.73	4.83	4.77	0.14	0.87
**Modified traditional process of sourdough production with lactic acid bacteria**	4.79	4.95	4.76	0.87	0.68

Age differences were compared by employing ANOVA. * Means sharing the same small letter in superscripts within a row are not significantly different (*p* > 0.05).

## Data Availability

Data will be made available on request.

## Supplementary Materials

The following supporting information can be downloaded at https://www.mdpi.com/article/10.3390/nu15214693/s1. File S1: Survey questionnaire.
