# Supplementary material for: FODMAPs—Unknowns for Consumers: First Survey in Serbia
Source: Nutrients. 2023 Nov 5;15(21):4693. doi: 10.3390/nu15214693 (PMC10648408; doi:10.3390/nu15214693)
Supplement: Supplementary file 1 [file nutrients-15-04693-s001.zip › nutrients-2654859-supplementary.pdf]

GutFriendlyCarbs

## SURVEY QUESTIONNAIRE

Dear Sir/Madam, before you is a questionnaire that was created for the purpose of collecting data and conducting research within the scientific research project called GutFriendlyCarbs, which is part of the IDEA Program, under the auspices of the Science Fund of the Republic of Serbia (Grant no. 7736059).

The survey is completely anonymous, and the data will be used exclusively for scientific purposes. We hope that you will take a few minutes of your time to fill out the questionnaire. Thank you in advance for your cooperation!

The subject of the survey research is the familiarity of the population of the Republic of Serbia with FODMAPs, their presence in bakery products, as well as the potential health problems they can cause.

FODMAP stands (is an acronym) for a group of nutrients- **F**ermentable **O**ligo- / **D**i- / **M**onosaccharides **A**nd **P**olyols**,** which are classified as carbohydrates and sugar alcohols (natural sweeteners such as sorbitol, xylitol, mannitol, etc.) which can cause gastrointestinal disorders associated with flatulence, pain in the intestines, intense gas production, diarrhea, etc. According to the latest findings, FODMAP compounds increase the symptoms of the following diseases: Irritable Bowel Syndrome (IBS), Crohn's disease, Celiac disease, Small Intestine Bacterial Overgrowth (SIBO) and etc.

### SOCIO-DEMOGRAPHIC CHARACTERISTICS

1. Gender:
   a) male b) female c) other (I don't want to declare)

2. Age: _______ years

3. Highest education:
   a) primary school b) secondary school c) undergraduate d) postgraduate (MSc/ PhD)

4. I am currently a resident of:
   a) rural areas b) smaller urban areas (up to 100,000 inhabitants) c) larger urban areas

5. Occupation:
   a) student b) company employee c) self-employed d) unemployed e) retired

6. Monthly income:
   a) below average (state level) b) average c) above average

### HEALTH EFFECTS OF FODMAP

1. Have you heard of the term FODMAP before this questionnaire?
   a) yes
   b) yes, but I don't know what it means and what effect they have on the body
   c) no

2. Do you have any of the symptoms of gastrointestinal disorder (Select all that apply)?

a) gluten intolerance
b) celiac disease
c) lactose intolerance
d) flatulence/excess gases
e) pains in the intestines
f) diarrhea
d) poor digestion/poor absorption of food
h) irritable bowel syndrome (IBS)
i) Crohn's disease
j) Small Intestine Bacterial Overgrowth (SIBO)
k) something else:_________________________
l) I have no symptoms of a gastrointestinal disorder

3. If you have any of the eating disorders, has the diagnosis been confirmed by a doctor?
a) yes (specify which _________________________) b) no c) I am in the testing phase

4. Are you on any kind of special diet:
a) gluten-free diet
b) lactose-free diet
c) reducing carbohydrates/keto diet
d) vegetarian/vegan diet
e) paleo diet
f) diet with increased content of dietary fiber
g) diet for diabetics
h) something else:___________________________________
i) I am not on a special diet

5. Do you use wholegrain flour products in your diet?
a) yes b) no

6. Do you use sweeteners in your diet?
a) yes b) no

7. If your answer to the previous question is "yes", what type of sweetener do you use? (Select all that apply)
a) aspartame
b) other synthetic sweeteners (saccharin, cyclamate, or thaumatin)
c) natural sweeteners (stevia, sorbitol, xylitol, mannitol, etc.)

8. Foods that contain significant amounts of FODMAP compounds are: (check the box with the statement you agree with)

| **Type of food products** | **Strongly disagree** | **Disagree** | **Neither agree nor disagree** | **Agree** | **Strongly agree** |
|---|---|---|---|---|---|
| Milk and dairy products | | | | | |
| Meat and meat products | | | | | |
| Fruits | | | | | |
| Nuts | | | | | |
| Vegetables | | | | | |

| | | | | | |
|---|---|---|---|---|---|
| Legumes | | | | | |
| Fish and seafood | | | | | |
| Cereals and bakery products | | | | | |
| Spices | | | | | |
| Alcoholic beverages | | | | | |
| Chocolates | | | | | |
| Candies | | | | | |
| Cookies | | | | | |
| Cakes | | | | | |

9. Cereal-based products that I consume to a significant extent are: (it is possible to circle more than one answer)
   a) bread b) pastries c) salty snacks and extruded products d) pasta e) cookies f) cakes g) breakfast cereals and expanded products (muesli/flakes)

## ATTITUDES REGARDING CEREAL-BASED PRODUCTS

1. Circle the number that best describes your agreement with the given statements:

| 1 Strongly disagree | 2 Disagree | 3 Somewhat disagree | 4 Neither agree nor disagree | 5 Somewhat agree | 6 Agree | 7 Strongly agree |
|---|---|---|---|---|---|---|

| **Perception of benefits/Usefulness** | | | | | | | |
|---|---|---|---|---|---|---|---|
| Eating Low-FODMAP bread would be beneficial for me | 1 | 2 | 3 | 4 | 5 | 6 | 7 |
| Eating Low-FODMAP pasta would be beneficial for me | 1 | 2 | 3 | 4 | 5 | 6 | 7 |
| Eating Low-FODMAP cookies would be beneficial for me | 1 | 2 | 3 | 4 | 5 | 6 | 7 |
| Eating Low-FODMAP cakes would be beneficial for me | 1 | 2 | 3 | 4 | 5 | 6 | 7 |
| Eating wholegrain bread would be beneficial for me | 1 | 2 | 3 | 4 | 5 | 6 | 7 |
| Eating wholegrain pasta would be beneficial for me | 1 | 2 | 3 | 4 | 5 | 6 | 7 |
| Eating wholegrain cookies would be beneficial for me | 1 | 2 | 3 | 4 | 5 | 6 | 7 |
| Eating wholegrain cakes would be beneficial for me | 1 | 2 | 3 | 4 | 5 | 6 | 7 |
| **Willingness to use** | | | | | | | |
| Would you be willing to use wholegrain bread in your diet? | 1 | 2 | 3 | 4 | 5 | 6 | 7 |
| Would you be willing to use wholegrain pasta in your diet? | 1 | 2 | 3 | 4 | 5 | 6 | 7 |
| Would you be willing to use wholegrain cookies in your diet? | 1 | 2 | 3 | 4 | 5 | 6 | 7 |
| Would you be willing to use wholegrain cakes in your diet? | 1 | 2 | 3 | 4 | 5 | 6 | 7 |
| Would you be willing to use Low-FODMAP bread in your diet? | 1 | 2 | 3 | 4 | 5 | 6 | 7 |
| Would you be willing to use Low-FODMAP pasta in your diet? | 1 | 2 | 3 | 4 | 5 | 6 | 7 |
| Would you be willing to use Low-FODMAP cookies in your diet? | 1 | 2 | 3 | 4 | 5 | 6 | 7 |
| Would you be willing to use Low-FODMAP cakes in your diet? | 1 | 2 | 3 | 4 | 5 | 6 | 7 |
| **Attitudes toward different FODMAP content reduction procedures** | | | | | | | |
| For the reduction of FODMAP content following, originated from natural sources, are used: enzymes (do not belong to the group of additives), baker's yeast, lactic acid bacteria (traditionally used in the production of dairy products), plant extracts and sprouted grains. | | | | | | | |

| | | | | | | | |
|---|---|---|---|---|---|---|---|
| I have a positive attitude towards the application of commercial purified enzymes (originating from microorganisms) in order to reduce the content of FODMAPs | 1 | 2 | 3 | 4 | 5 | 6 | 7 |
| I have a positive attitude about the use of plant extracts in order to reduce the content of FODMAPs | 1 | 2 | 3 | 4 | 5 | 6 | 7 |
| I am positive about reducing FODMAPs by using sprouted grains | 1 | 2 | 3 | 4 | 5 | 6 | 7 |
| I have a positive attitude about reducing FODMAPs with a modified standard manufacturing process using baker's yeast | 1 | 2 | 3 | 4 | 5 | 6 | 7 |
| I am positive about FODMAP reduction using a modified traditional sourdough production process using lactic acid bacteria | 1 | 2 | 3 | 4 | 5 | 6 | 7 |
| **Attitudes about health needs in nutrition** | | | | | | | |
| I pay attention to a healthy diet | 1 | 2 | 3 | 4 | 5 | 6 | 7 |
| Adequate intake of dietary fiber in my diet is important to me | 1 | 2 | 3 | 4 | 5 | 6 | 7 |
| I pay special attention to "gut health" | 1 | 2 | 3 | 4 | 5 | 6 | 7 |
| I pay special attention to digestive problems | 1 | 2 | 3 | 4 | 5 | 6 | 7 |
| **Self-perception** | | | | | | | |
| I consider myself a health-conscious person | 1 | 2 | 3 | 4 | 5 | 6 | 7 |

2. Rate the importance of certain characteristics when choosing/purchasing cereal-based products, by circling a number on a scale from 1 to 6, where the values are presented as:

1- Very unimportant
2- Unimportant
3- Slightly unimportant
4- Slightly important
5- Important
6- Very important

| Characteristics | Bread | Pasta | Cookie | Cakes |
|---|---|---|---|---|
| **The price** | 1-2-3-4-5-6 | 1-2-3-4-5-6 | 1-2-3-4-5-6 | 1-2-3-4-5-6 |
| **Taste** | 1-2-3-4-5-6 | 1-2-3-4-5-6 | 1-2-3-4-5-6 | 1-2-3-4-5-6 |
| **Health impact** | 1-2-3-4-5-6 | 1-2-3-4-5-6 | 1-2-3-4-5-6 | 1-2-3-4-5-6 |
| **Appearance/texture** | 1-2-3-4-5-6 | 1-2-3-4-5-6 | 1-2-3-4-5-6 | 1-2-3-4-5-6 |
| **Being new** | 1-2-3-4-5-6 | 1-2-3-4-5-6 | 1-2-3-4-5-6 | 1-2-3-4-5-6 |
| **Being traditional** | 1-2-3-4-5-6 | 1-2-3-4-5-6 | 1-2-3-4-5-6 | 1-2-3-4-5-6 |

## HABITS REGARDING THE PURCHASE OF CEREAL-BASED PRODUCTS: BREAD

1. How often do you buy bread?
   a) daily b) minimally twice a week c) less than twice a week d) I don't eat bread

2. Where do you most often buy bread?
   a) at the bakery b) at the grocery store c) I don't buy it, I make it myself

3. What kind of flour do you buy/make bread from? (Select all that apply)
   a) wheat b) spelt c) rye d) corn e) buckwheat
   f) oat d) from a ready-made gluten-free mixture
   f) other (write) ________________________________

4. What type of bread do you consume/make?
   a) bread with yeast b) unleavened bread c) bread made from sprouted grains

d) sourdough bread e) bread with a leavening agent (baking powder, baking soda) f) other (write) ______________________________

5. What bread do you buy/ often make?
   a) white / semi-white bread b) bread made from wholegrain flour c) bread made from several types of cereals
   d) gluten-free bread e) other (write) ______________________________

6. I eat bread: (Select all that apply)
   a) for breakfast b) for lunch c) for dinner d) for snack

7. How much bread do you eat DAILY?
   a) < 100 g (~ 3 slices) b) 100 – 200 g (3 - 6 slices) c) > 200 g

## PASTA

8. How often do you eat pasta?
   a) daily b) several times a week c) several times a month
   d) several times a year e) I don't consume pasta

9. How much pasta (cooked, without a side dish) do you eat WEEKLY?
   a) < 150 g (~1 portion) b) 150 – 450 g c) > 450 g

10. I most often buy pasta at:
    a) the grocery store b) the market c) I don't buy it, I make it myself

11. What kind of flour do you buy/make pasta from? (it is possible to circle multiple answers)
    a) common wheat b) durum wheat c) corn
    d) spelt e) buckwheat f) rice d) other (write) ___________________________

12. I consume pasta from:
    a) white flour b) wholegrain flour

## COOKIES

13. How often do you consume cookies and related products?
    a) daily b) several times a week c) several times a month
    d) several times a year e) I don't consume cookies

14. I most often buy cookies and related products at:
    a) the grocery store b) I don't buy them, I make them myself

15. I buy/make cookies from:
    a) wheat flour b) gluten-free flour c) oat flour d) rye flour
    e) spelt flour f) other (write-in) ___________________________

16. I consume cookies made with:
    a) white flour b) wholegrain flour

## CAKES

17. How often do you consume cakes and confectionery products?
    a) daily b) several times a week c) several times a month
    d) several times a year e) I don't eat cookies

18. Most often I buy cakes at:
    a) the grocery store b) the market
    c) the specialized stores d) I don't buy them, I make them myself

19. I buy/make cakes from:
    a) wheat flour b) wholegrain wheat flour c) oat flour d) gluten-free flour
    e) other (write) ___________________________

COMMENT:
___________________________________________________________________________

Thank you for your participation!
